# Transcriptome analysis of differentially expressed genes in rice seedling leaves under different nitrate treatments on resistance to bacterial leaf blight

**DOI:** 10.3389/fpls.2024.1436912

**Published:** 2024-07-04

**Authors:** Xintong Liu, Shunquan Chen, Changjian Miao, Huijing Ye, Qingchao Li, Hongzhen Jiang, Jingguang Chen

**Affiliations:** ^1^ School of Agriculture and Biotechnology, Sun Yat-sen University, Shenzhen, Guangdong, China; ^2^ Shenzhen Institute of Molecular Crop Design, Shenzhen, Guangdong, China; ^3^ Bijie Academy of Agricultural Sciences, Bijie, Guizhou, China

**Keywords:** nitrate, bacterial leaf blight, transcriptome sequencing, differentially expressed genes, rice

## Abstract

Nitrogen (N), as one of the most abundant mineral elements in rice, not only is the primary limiting factor for rice yield, but also impacts plant disease resistance by modulating plant morphology, regulating biochemical characteristics, as well as enhancing metabolic processes. Bacterial blight, a severe bacterial disease caused by *Xanthomonas oryzae* pv. *oryzae* (*Xoo*), significantly impairing rice yield and quality. Previous studies have shown that moderate application of nitrate nitrogen can improve plant disease resistance. However, further exploration is urgently required to investigate the involvement of the nitrate nitrogen signaling pathway in conferring resistance against bacterial leaf blight. In this study, we employed transcriptome sequencing to analyze the differentially expressed genes under various concentrations of nitrate supply duringrice bacterial blight infection. Our research reveals that nitrate nitrogen supply influences rice resistance to bacterial leaf blight. Through transcriptomic profiling of rice leaves inoculated under different nitrate nitrogen concentrations, we identified 4815 differentially expressed genes (DEGs) among four comparison groups, with notable differences in DEG enrichment between low and high nitrate nitrogen conditions, with some members of the NPF family implicated and we preliminarily elucidated the molecular regulatory network in which nitrate nitrogen participates in bacterial leaf blight resistance. Our findings provide a novel insight into a mechanism involving the nitrate nitrogen drive wider defense in rice.

## Introduction

Rice, *Oryza sativa* L, holds great significance as one of the world’s foremost staple crops, contributing to a quarter of global grain production. More than 50% of the global population relies on rice as their primary food source. In China, rice cultivation covers approximately 30 million hectares, making it the largest producer worldwide. Nitrogen (N), being among the most abundant mineral elements in rice, serves as a primary determinant of yield. The application of nitrogen fertilizer has significantly contributed to global rice production but has also resulted in increasingly severe economic and environmental challenges. Excessive use of nitrogen fertilizer leads to resource wastage, environmental pollution, and a decline in rice quality (Hou et al., 2021). Therefore, there is an urgent comprehension of the molecular regulatory mechanisms underlying nitrogen uptake and utilization in rice for developing strategies aimed at enhancing nitrogen use efficiency (NUE) (Li et al., 2017). NUE plays a crucial role in achieving high-yielding and high-quality rice production. Nitrogen, being one of the most abundant elements in living organisms, serves various physiological functions within plants while interacting with both plants and pathogens. Different forms of nitrogen exert diverse effects on host plants and pathogen development, thereby influencing disease severity and resistance (Huber and Watson, 1974). Nitrogen impacts plant disease resistance by modulating plant morphology, regulating biochemical characteristics, as well as enhancing metabolic processes. Upon pathogen invasion, different forms of nitrogen exhibit distinct influences on plant disease resistance. Under nitrate conditions, NO signaling compounds such as salicylic acid and polyamines are upregulated. In contrast to nitrate-nitrogen effects, ammonium-nitrogen application reduces these defense signal components while increasing nutrient sources for pathogens like soluble sugars amino acids, and γ-aminobutyric acid. Thus, nitrate-nitrogen enhances plant disease resistance (Mur et al., 2017).

Bacterial leaf blight, induced by *Xanthomonas oryzae* pv*.oryzae* (*Xoo*) is a highly prevalent and devastating bacterial disease that affects rice globally. It is particularly prominent in major rice-growing regions such as East China, South China, and Central China with its severity escalating in recent years (Liu et al., 2014). The pathogen infiltrates through stomata and wounds on rice leaves leading to distinct yellow-brown or gray-white lesions that pose significant threats to rice yield. In cucumber, compared to ammonium, nitrate treatment significantly alleviated the levels of wilting acid, membrane oxidation, and organelle damage in cucumber leaves under *Fusarium oxysporum* f.sp.*cucumerinum* (FOC) infection conditions by increasing the rate of photorespiration and metabolic processes. Related metabolomics and biochemical tests conducted through exogenous application of isoniazid (INH), a photorespiration inhibitor, further confirmed the crucial role of nitrate-mediated photorespiration in cucumber’s disease resistance (Sun et al., 2021). In a previous study, researchers identified the ammonium transporter protein AMT1;1 in rice. This protein plays a crucial role in mediating NH_4_^+^ accumulation, nitrogen metabolism, and ethylene signaling to enhance the defense against sheath blight disease. The findings of this investigation unveil a novel mechanism by which AMT1;1 boost rice resistance to sheath blight disease, emphasizing the significance of the nitrogen assimilation signaling pathway in understanding the molecular mechanisms underlying disease resistance. Nevertheless, urgent further exploration is necessary to investigate the involvement of the nitrate nitrogen signaling pathway in conferring resistance against bacterial leaf blight (Wu et al., 2022).

Nitrate nitrogen, as one of the primary forms of nitrogen uptake in rice, is involved in multiple biological processes essential for its growth and development. However, research on the involvement of nitrate nitrogen in the resistance mechanism against bacterial leaf blight in rice is limited. This knowledge gap hinders our ability to optimize nitrogen supply and improve rice yield by enhancing its resistance against bacterial leaf blight. In this study, we employed transcriptome sequencing to analyze the differentially expressed genes under various concentrations of nitrate supply during rice bacterial blight infection, aiming to elucidate the molecular regulatory network of nitrate nitrogen in rice resistance against bacterial blight disease and deepen our understanding of its mechanisms.

## Materials and methods

### Plant material and growth conditions

The rice varieties used were *Oryza sativa* L. ssp. *Japonica* cv. ‘Nipponbare’ which is the modern cultivars of the typical japonica subspecies for low pathogen tolerance. The seeds were germinated in soaking bags under dark conditions. After soaking for 3 days at 37°C, germination was initiated. Once the seeds sprouted, those with consistent growth were transferred to a 96-well plate filled with distilled water. The water was changed every 3 days during this period. After 7 days, it was replaced with a 1/8 strength IRRI nutrient solution, followed by a switch to a 1/4 strength solution after another 3 days. Subsequently, a half-strength IRRI nutrient solution was used. The cultivation conditions are as follows: lighting at 30°C for 14 hours, followed by darkness at 28°C for 10 hours; maintaining a humidity level of 60%; and providing an illumination intensity of 18000 Lux.

After growing for 2 weeks, seedlings were divided into 4 groups and different concentrations of nitrogen treatments were applied to the rice plants. Other elements in the nutrient solutions remained at the same concentration as the full-strength IRRI solution throughout the experiment. The treatment duration was one week and the nitrogen source concentrations were as follows: low nitrate (0.5 mM NO_3_^-^), normal nitrate (2.5 mM NO_3_^-^) and high nitrate (5 mM NO_3_^-^). After treated for 14 days, seedlings were ready to be inoculated. Each group within each nitrogen concentration treatment was divided into two groups: an inoculation group (IN) and a control check group (CK). The *Xanthomonas oryzae* pv*. oryzae* pathogen (*Xoo* strain PXO99A) was cultivated on PSA medium, and after 48h of cultivation at 28°C, all bacteria were collected in sterile water and suspended to an OD=1.0 for inoculation. The leaves of rice plants in the inoculation group were subjected to inoculation experiments by leaf-clipping method, while continuous observations on phenotypes were conducted after inoculation.

### Phenotype measurement and analysis

After 3 weeks of inoculation, 12 randomly selected rice plants from each group were used to record the length of disease lesions and their phenotypes were photographed. Meanwhile, leaf samples were taken from each group with 3 replicates, where each replicate consisted of a mixture of 10 leaves. The samples were stored at -80°C for subsequent RNA-seq analysis. The phenotypic data were averaged and subjected to multiple comparison analysis using the LSD method with a significance level of *P* < 0.05.

### Isolation of total RNA, cDNA library construction, and illumine sequencing

Total RNA extraction was extracted using Invitrogen TRIzol. The quality of the total RNA was assessed in two parts: concentration and purity were measured using a nanodrop Thermo Scientific NanoDrop 2000 (Thermo Scientific, Waltham, Massachusetts, USA), while integrity was evaluated by RNA-specific agarose gel electrophoresis and Agilent 2100 Bioanalyzer with the RNA 6000 Nano kit 5067–1511 (Agilent Technologies Inc, California, USA). Total RNA samples with a quantity greater than 1 microgram were used to generate cDNA libraries using the NEB Next Ultra II RNA Library Prep Kit for Illumina (New England Biolabs Inc; Ipswich, Massachusetts, USA) with the strand-specific library preparation kit NEB Next Ultra Directional RNA Library Prep Kit for Illumina. The library concentration was determined by Pico green assay (Quantifluor-ST fluorometer, Promega, Madison, Wisconsin, USA E6090; Quant-iT PicoGreen dsDNA Assay Kit, Invitrogen California USA P7589), and effective library concentrations were quantified by QPCR on Step One Plus Real Time PCR Systems (Thermo Scientific Waltham Massachusetts USA). A total of twelve libraries were constructed and sequenced on an Illumina platform using PE150 mode after being gradually diluted and quantified following DNA library acquisition.

### Data filtering, mapping and control quality

After sequencing, the raw data obtained from sequencing will undergo certain filtering and quality control. The software fastp (0.22.0) (Chen et al., 2018) will be used to filter out sequences with adapters and remove reads below Q20. The data after quality control will be used for subsequent analysis.

### Differentially expressed genes

HTSeq (v0.9.1) (Anders et al., 2014) was used to count the Read Count values aligned to each gene, which were considered as the expression level of genes. To ensure comparability of gene expression levels among different genes and samples, FPKM (Fragment Per Kilo bases per Million fragments) was used for normalization. Based on the gene’s FPKM value, DESeq2 (Love et al., 2014) was employed for differential expression analysis between two comparison groups by setting criteria for differentially expressed genes: fold change in expression |log_2_FoldChange| > 1 and *P* -value < 0.05.

### Gene ontology enrichment and KEGG pathway enrichment analysis

Performing GO enrichment analysis using topGO (v2.50.0) (Alexa et al., 2006), the *P* -values are calculated using the hypergeometric distribution method, with a significance threshold set at *P*-value < 0.05, to identify significantly enriched GO terms for differentially expressed genes (all/up/down). This helps determine the main biological functions performed by these differentially expressed genes. For KEGG pathway enrichment analysis, clusterProfiler software (v4.6.0) (Wu et al., 2021) is used, focusing on significantly enriched pathways with a *P*-value < 0.05. Gene Set Enrichment Analysis (GSEA) software (v4.1) (Subramanian et al., 2005) is utilized for gene set enrichment analysis without requiring specific differential gene values; instead, all genes are ranked based on their differential expression levels between two sample groups and statistical methods are employed to test whether predefined gene sets are enriched at the top or bottom of the ranked list.

### Validation of gene expression

To validate the reliability of the transcriptome sequencing results, 14 differentially expressed genes (DEGs) were randomly selected from rice transcriptome data for qRT-PCR verification. For each gene, the measurements were performed three independent times. The rice β-actin gene (*LOC_Os03g50885*) was used as an internal reference gene, and primer sets were designed using ncbi primer-blast (https://www.ncbi.nlm.nih.gov/tools/primer-blast/), listed in **Supplementary Table S1**. Reverse transcription and qPCR were performed using HiScript^®^ III RT SuperMix for qPCR (+gDNA wiper) Vazyme and BioEasy SYBR Green I Real Time PCR Kit. The 10 μl PCR mixture consisted of 5μl 2x SYBR Mix, 0.2μM of each Primers, 0.06 μl Taq DNA Polymerase and diluted cDNA. The PCR program comprised the following steps: 94°C, 2min; 94°C, 10s, 60°C, 15s, 72°C, 30s, 40 cycles. The second dissociation stage was conducted as follows: 95°C, 2min; 72°C, 1min; 95°C, 30s, with increments of 0.5°for 1s; 30°C,1min. The results were calculated using the 2^-ΔΔCT^ method (Livak and Schmittgen, 2001).

## Results

### Phenotype of seedlings after *Xoo* inoculated treatment

**Figure 1:**
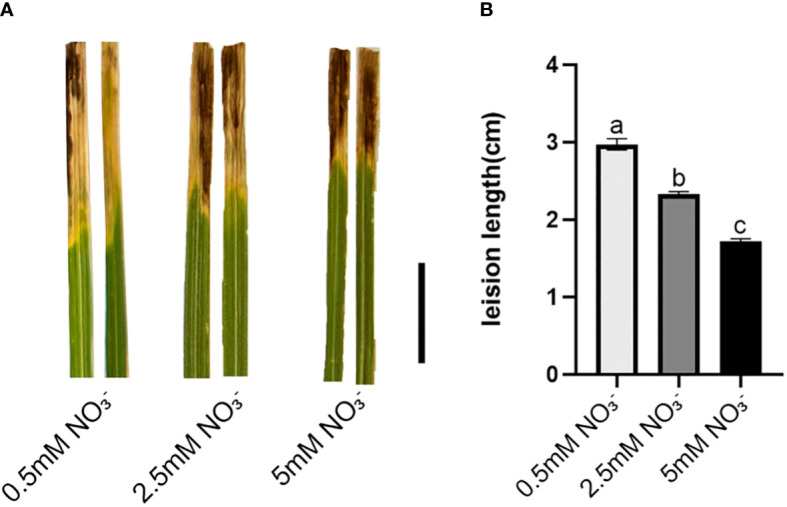
Leaves inoculated with *Xoo* strain *PXO99A* under different concentrations of nitrate supplies. **(A)** Phenotyping after 21 days of exposure to different concentrations of nitrate supply, Bar=1cm. **(B)** Average lesion length after 21 days of exposure to different concentrations of nitrate supply. Values are mean ± SD (n=10). Different letters show significant differences at *P* < 0.05 (one-way ANOVA).

To investigate the impact of varying concentrations of nitrate as a nitrogen source on the resistance of rice plants against the pathogen responsible for bacterial leaf blight, this study assessed changes in resistance by measuring the length of disease lesions on rice leaves following inoculation experiments. The lesion length of leaves was calculated. As shown in the **Figure 1**, compared to low nitrate supply (LN), the lesion length of leaves shortened by 19.56% and 42.24% for normal nitrate supply (NN) and high nitrate supply (HN), respectively. Moreover, the phenotypic results demonstrate that an increased concentration of nitrate supply enhances rice resistance against *Xoo*, with the most prominent disparity observed between the low-nitrate and high-nitrate groups. Consequently, these two nitrate concentration groups were selected for subsequent RNA-seq analysis.

### Overview of transcriptome quality assessment

In this study, a total of 12 transcriptome libraries were constructed using the inoculated leaves for 3 weeks under low nitrate supply (0.5 mM NO_3_^-^, LN) and high nitrate supply (5 mM NO_3_^-^, HN), along with their respective controls. The samples included the LNin group (rice leaves inoculated with low nitrate and inoculation) and its control group (LNck), as well as the HNin group (rice leaves inoculated with high nitrate and inoculation) and its control group (HNck), each consisting of two or three replicates.

After sequencing 12 samples, a total of 56.48 Gb of raw reads were obtained. The average number of raw reads for the HNin group was 47920338, for the HNck group was 4980524, for the LNin group was 48792030, and for the LNck group was 41707552 (**Table 1**). To ensure accurate subsequent information analysis without interference factors present in the data set, we performed adapter trimming and removed reads with quality scores below Q20. Consequently, a total of 53.41 Gb of clean data were obtained with an average of 4.45 Gb per sample. The average Q30 value across all groups reached an impressive level of 94%. Furthermore, the average number of clean reads per sample were as follows: LNck 3944718, LNin 46123427, HNck 47155097, HNin 45309072 (**Table 1**). The filtered clean reads were then aligned to the Nipponbare genome (GCF_001433935.1_IRGSP-1.0_genomic) using HISAT2 from NCBI database. The overall alignment rate (Total Mapped) ranged from 91.92% to 96.42%, while the uniquely mapped rate (Uniquely Mapped) ranged from95.01% to96.70%. Additionally, the multiple mapped rate (Multiple Mapped) varied between3.30% and4.99% (**Table 1**). These results demonstrate that both the quality and quantity aspects of our sequencing approach are satisfactory and provide a solid foundation for further bioinformatics analysis.

**Table 1:** Summary of RNA-seq data and reads mapping.

Sample	Raw reads	Clean reads	Total mapped reads	Q20(%)	Q30(%)	Total_Mapped	Multiple_Mapped	Uniquely_Mapped
LNck1	42062304	39824750	38270036 (96.10%)	97.86	94.08	38270036 (96.10%)	1534890 (4.01%)	36735146 (95.99%)
LNck2	40202426	37980846	36078346 (94.99%)	97.96	94.44	36078346 (94.99%)	1646348 (4.56%)	34431998 (95.44%)
LNin1	54374226	51405494	47943292 (93.26%)	97.75	93.82	47943292 (93.26%)	1736383 (3.62%)	46206909 (96.38%)
LNin2	50177124	47411162	43993978 (92.79%)	97.83	94.03	43993978 (92.79%)	2193514 (4.99%)	41800464 (95.01%)
LNin3	41824740	39553624	36355900 (91.92%)	97.74	93.81	36355900 (91.92%)	1247719 (3.43%)	35108181 (96.57%)
HNck1	44258126	41884066	40383532 (96.42%)	98.17	94.82	40383532 (96.42%)	1390394 (3.44%)	38993138 (96.56%)
HNck2	50862464	48111006	46061142 (95.74%)	97.7	93.7	46061142 (95.74%)	1777260 (3.86%)	44283882 (96.14%)
HNin1	48626678	45969506	44137665 (96.02%)	97.62	93.48	44137665 (96.02%)	1530055 (3.47%)	42607610 (96.53%)
HNin2	49324554	46619360	44499096 (95.45%)	97.79	93.91	44499096 (95.45%)	1467799 (3.30%)	43031297 (96.70%)

### Correlation analysis

As the correlation of gene expression levels between samples is a crucial indicator for evaluating sample selection and an essential prerequisite for conducting differential expression analysis, we computed Pearson’s correlation coefficients based on FPKM values of all genes within each sample. A higher correlation coefficient signifies a stronger resemblance in expression patterns among samples. Through association analysis, we identified biological replicates within each group with correlation coefficients exceeding 0.97 (**Supplementary Figure S1**), indicating excellent reproducibility of biological replicates and facilitating subsequent analyses.

### Differentially expressed genes identified using RNA-seq

To investigate the molecular mechanisms underlying rice resistance to white leaf disease under varying nitrate supply concentrations, this study employed DESeq2 for differential gene expression analysis. Differential genes were selected based on the following criteria: |log_2_(FoldChange)|>1, *P*-value<0.05. A total of 4815 differentially expressed genes (DEGs) were identified, with 2511 upregulated in at least one group and 3064 downregulated in at least one group (**Supplementary Figure S2**). HNck was compared to LNck, resulting in the identification of 516 upregulated genes and 731 downregulated genes (**Supplementary Table S2**). HNin was compared to LNin, leading to the identification of 523 upregulated genes and 1473 downregulated genes (**Supplementary Table S3**). Comparing LNin with LNck revealed 1763 upregulated genes and 1258 downregulated genes (**Supplementary Table S4**). When comparing HNin with HNck, we detected 503 upregulated genes and 886 downregulated genes (**Supplementary Table S5**) Interestingly, a higher number of differentially expressed genes (DEGs) were identified as upregulated under low nitrate supply; however, under high nitrate supply, more DEGs were observed as downregulated. These two groups of DEGs exhibited distinct response patterns. Furthermore, when comparing LNin with HNin, a significantly greater number of DEGs were found as downregulated than upregulated. This suggests that rice exhibits differential resistance mechanisms against bacterial blight disease under low and high nitrate supplies leading to phenotypic differences.

In order to further investigate the disparities in differentially expressed genes (DEGs) within rice plants following nitrate inoculation at these two concentrations, we conducted additional analysis on the identified DEGs between these two comparison groups. Our findings revealed a total of 279 upregulated DEGs and 453 downregulated DEGs that were concurrently present in both groups (**Supplementary Figure S2**). Moreover, we observed 39 DEGs exhibiting contrasting expression patterns between these two groups: specifically, 27 genes were upregulated in the low-nitrate (LN) inoculated group but downregulated in the high-nitrate (HN) inoculated group; conversely, 12 genes displayed an inverse pattern (**Supplementary Figure S2**).

**Figure 2:**
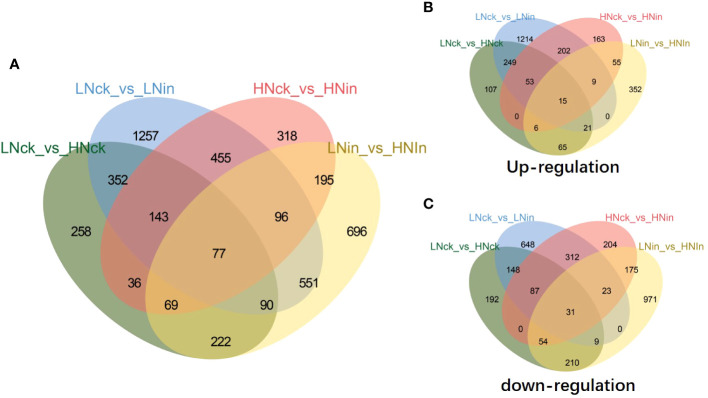
Venn diagrams for DEGs in response to different nitrate concentrations and inoculation. **(A)** All DEGs. **(B)** Upregulated genes. **(C)** Downregulated genes. LN: low nitrate supply (0.5 mM NO_3_^-^); HN: high nitrate supply (0.5 mM NO_3_^-^); ck: without inoculation; in: inoculation.

From the Venn diagram, it can be observed that there are 77 DEGs shared among these four comparison groups (**Figure 2**). Further analysis reveals that out of these, 15 genes are upregulated in all four groups respectively. They are as follows: EP1-like glycoprotein 2 gene (*LOC_Os01g72810*); probable calcium-binding protein CML31 gene (*LOC_Os01g72530*); expressed protein gene (*LOC_Os03g07200*); E3 ubiquitin-protein ligase SRFP1-like gene (*LOC_Os03g22680*); hapless 2 gene (*LOC_Os03g51350*); ABC transporter B family member 19 gene (*LOC_Os04g38570*); alpha-galactosidase 3 gene (*LOC_Os07g48160*); aspartic proteinase CDR1 gene (*LOC_Os08g10670*); U-box domain-containing protein 19 gene (*LOC_Os09g21120*); acyltransferase-like protein At1g54570 gene (*LOC_Os09g33530*); glutelin type-D 1-like gene (*LOC_Os09g37976*); vegetative cell wall protein gp1 gene (*LOC_Os11g06150*); probable glycerol-3-phosphate acyltransferase 3 gene (*LOC_Os11g45400*); malate synthase-like gene (*LOC_Os04g40990*); Hypothetical conserved gene (*LOC_Os08g08710*); 31 genes are downregulated in all four groups, such as: receptor-like serine/threonine-protein kinase SD1–8 gene (*LOC_Os01g57560*); protein THYLAKOID RHODANESE-LIKE, chloroplastic-like gene *(LOC_Os02g15750*); photosystem II 5 kDa protein, chloroplastic gene (*LOC_Os02g37060*);UPF0603 protein chloroplastic-like gene (*LOC_Os05g33280*); 15-cis-phytoene desaturase, chloroplastic/chromoplastic gene (*LOC_Os07g43370*); probable glutathione S-transferase GSTU6 gene (*LOC_Os10g38710*); Among these genes, certain ones have drawn our attention due to their differential expression under various treatments (**Figure 2**). For instance, *OsWRKY27* (*LOC_Os01g40430*) was found to be upregulated in LNck-LNin and HNck-HNin conditions but downregulated in LNin-HNin condition. This suggests that inoculation induces the expression of this gene, with higher expression observed under low nitrate supply compared to high nitrate supply. On the other hand, *OsRLR1* (*LOC_Os10g07978*) showed upregulation only in HNck-HNin condition, indicating that inoculation specifically induces its upregulation under high nitrate supply. Lastly, *OsbHLH6* (*LOC_Os04g23550*) was found to be upregulated in LNck-LNin but downregulated in HNck-HNin and LNin-HNin conditions (**Figure 2**). This implies that inoculation significantly reduces the expression of this gene under high nitrate supply.

**Figure 3:**
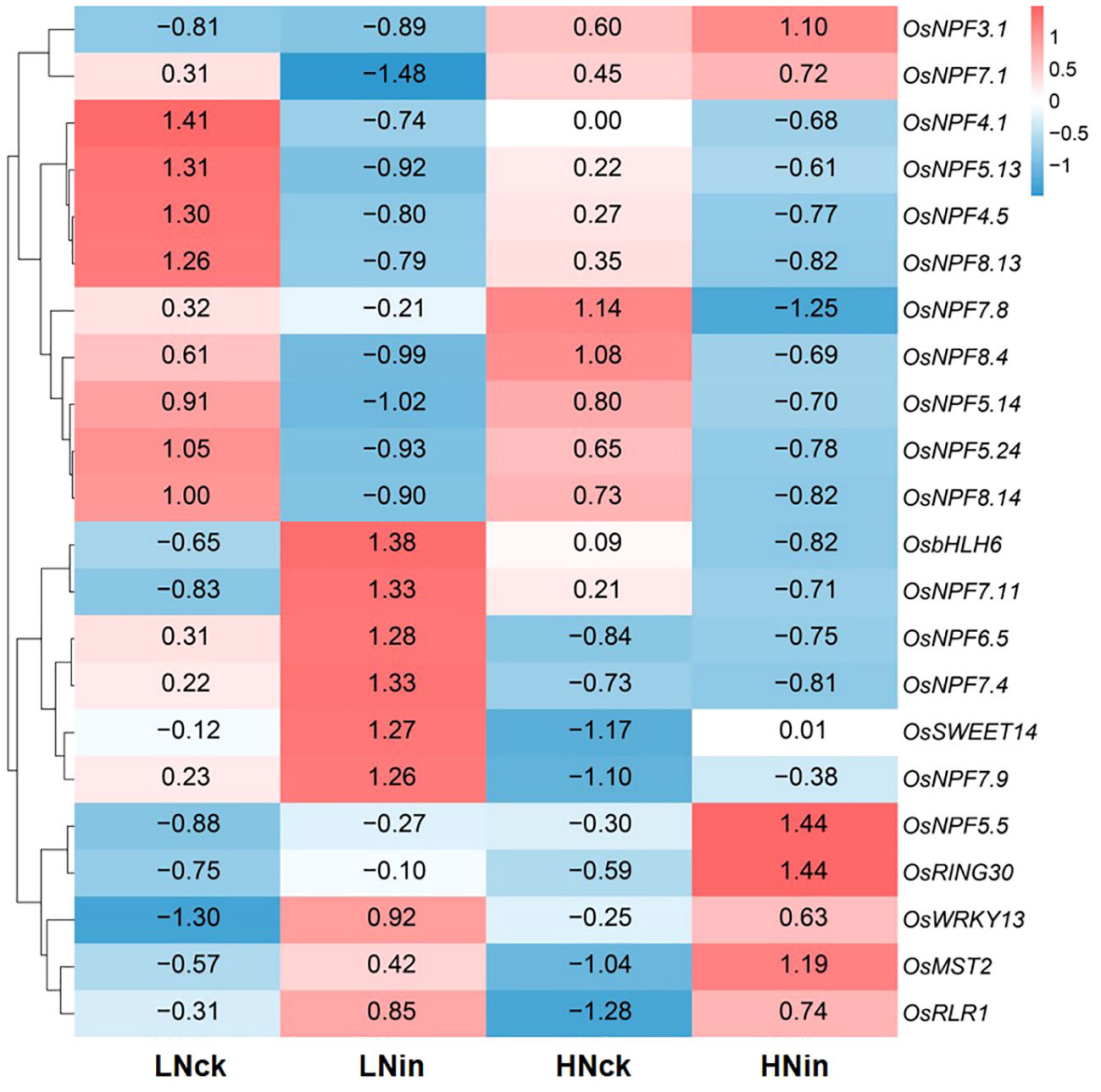
Heat map of the DEGs.

Simultaneously, we employed Pheatmap to analyze the expression levels of the same gene across different samples and the expression patterns of diverse genes within the same sample. Distances were calculated using the Euclidean method, and hierarchical clustering analysis was conducted using Complete Linkage. As shown in **Figure 3**, these DEGs could be classified into four distinct groups based on their evolutionary tree. The first group exhibited higher expression levels in both control groups LNck and HNck, suggesting that most genes in this category may be responsive to nitrogen. The second group displayed elevated expression primarily in HNck and HNin, indicating that genes in this class might be induced by high nitrate concentrations. The third group demonstrated increased expression levels in LNin and LNck, implying that genes within this cluster may be stimulated by low nitrate concentrations. Lastly, the fourth group showed heightened expression levels in both inoculated groups LNin and HNin, suggesting that genes belonging to this category might be induced by inoculation. In terms of global transcriptome profiles, LNck exhibited similarities with HNck while LNin shared resemblances with HNin regarding their respective expression patterns. This suggests that under identical nitrate supply concentrations, differences arising from inoculation can lead to divergent gene expressions within rice plants; even when variations exist in nitrate supply concentration, relatively similar gene expressions can persist between groups with or without inoculation.

### GO enrichment

**Figure 4:**
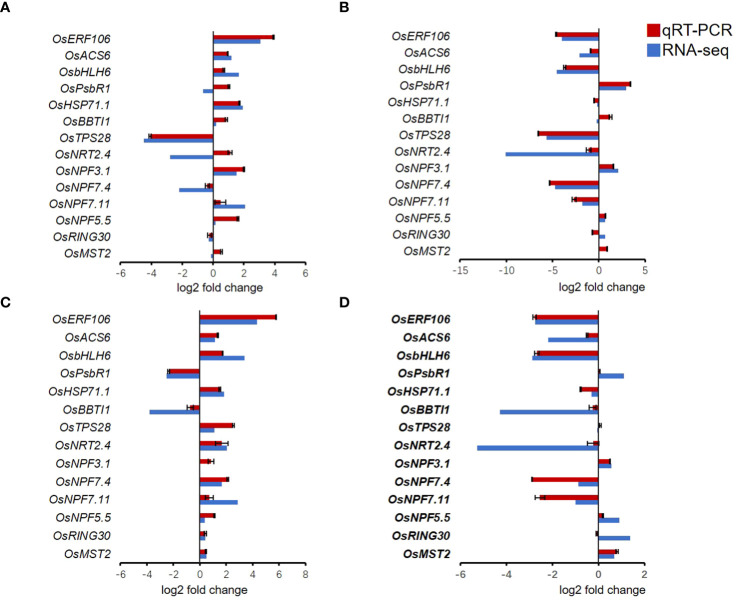
GO enrichment analysis of the DEGs. **(A)** LNck-HNck. **(B)** LNin-Hnin. **(C)** HNck-Hnin. **(D)** HNck-Hnin.

To further investigate the differential response patterns of rice to *Xoo* under varying nitrate supply, we performed Gene Ontology (GO) enrichment analysis on DEGs. A total of 289 unique GO terms were significantly enriched, comprising 169 in biological processes (BP), 53 in cellular components (CC), and 67 in molecular functions (MF) (**Figure 4**). Among the successfully enriched DEGs, there were 1686 up-regulated genes and 2098 down-regulated genes. The top five frequently enriched up-regulated genes included amino acid permease family protein (*LOC_Os01g71740*), amino acid permease family protein (*LOC_Os01g71760*), amino acid permease family protein (*LOC_Os01g42234*), amino acid permease family protein (*LOC_Os01g71700*), and OsNYC1 (*LOC_Os01g12710*). The top five frequently enriched down-regulated genes comprised OsLHCB5 (*LOC_Os11g13890*), chlorophyll A-B binding protein (*LOC_Os04g38410*), chlorophyll A-B binding protein (*LOC_Os01g52240*), chlorophyll A-B binding protein (*LOC_Os01g41710*), and chlorophyll A-B binding protein (*LOC_ Os06g21590*) (**Figure 4**). The significance threshold for enrichment was set at FDR ≤ 0.05.

For the control group LNck-HNck (**Figure 4A**), a total of 70 GO terms were significantly enriched, comprising 22 BP, 35 CC, and 13 MF. The differentially DEGs exhibited significant enrichment in GO terms closely associated with chloroplasts, such as chloroplast part (GO: 0044434, CC), plastid part (GO: 0044435, CC), thylakoid (GO: 0009579, CC), thylakoid part (GO: 0044436, CC), photosynthesis (GO: 0015979, BP), chloroplast thylakoid (GO: 0009534, CC), plastid thylakoid (GO: 0031976, CC), chloroplast thylakoid membrane (GO: 0009535, CC), plastid thylakoid membrane (GO: 0055035, CC), chloroplast stroma (GO: 0009570, CC), photosynthetic membrane (GO: 0034357, CC), thylakoid membrane (GO: 0042651, CC), plastid stroma (GO: 0009532, CC), chloroplast (GO: 0009507, CC), chloroplast ribulose bisphosphate carboxylase complex (GO: 0009573, CC), ribulose bisphosphate carboxylase complex (GO: 00 48492, CC), oxidation-reduction process (GO: 0055114, BP), reductive pentose-phosphate cycle (GO: 0019253, BP), photosynthesis, dark reaction (GO: 0019685, BP). Furthermore, the DEGs demonstrated oxidoreductase activity (GO: 0016491, MF).

For the inoculation group LNin-HNin (**Figure 4B**), a total of 111 GO terms were significantly enriched, including 65 BP, 29 CC, and 17 MF. The DEGs, showed significant enrichment in chloroplast (GO: 0009507, CC), plastid (GO: 0009536, CC), plastid part (GO: 0044435, CC), chloroplast part (GO:0044434, CC), thylakoid (GO:0009579, CC), thylakoid part (GO: 0044436, CC), plastid stroma (GO: 0009532, CC), chloroplast thylakoid (GO: 0009534, CC), plastid thylakoid (GO: 0031976, CC), oxidation-reduction process (GO: 0055114, BP), chloroplast stroma (GO: 0009570, CC), thylakoid membrane (GO: 0042651, CC), oxidoreductase activity (GO: 0016491, MF), photosynthetic membrane (GO: 0034357, CC), chloroplast thylakoid membrane (GO: 0009535, CC), plastid thylakoid membrane (GO: 0055035, CC), generation of precursor metabolites and energy (GO: 0006091, BP), photosynthesis (GO: 0015979, BP), amino sugar metabolic process (GO: 0006040, BP), aminoglycan catabolic process (GO: 0006026,BP).

The LN inoculation group LNck-LNin (**Figure 4C**), exhibited significant enrichment in a total of 182 GO terms, including 101 BP, 26 CC, and 55 MF. Notably, the DEGs demonstrated remarkable in intrinsic component of membrane (GO: 0031224, CC), integral component of membrane (GO: 0016021, CC), membrane part (GO: 0044425, CC), membrane (GO: 0016020, CC), protein serine/threonine kinase activity (GO: 0004674, MF), photosystem I (GO: 0009522, CC), plasma membrane (GO: 0005886, CC), photosystem (GO: 0009521, CC), protein kinase activity (GO: 0004672, MF), protein phosphorylation (GO: 0006468, BP), cell periphery (GO: 0071944, CC), tetrapyrrole binding (GO: 0046906, MF), defense response (GO: 0006952, BP), photosynthetic membrane (GO: 0034357, CC), pigment binding (GO: 0031409, MF), phosphotransferase activity, alcohol group as acceptor (GO: 0016773, MF), photosynthesis, light harvesting in photosystem I (GO: 0009768, BP), photosynthesis (GO: 0015979, BP), thylakoid part (GO: 0044436, CC), transcription regulator activity (GO: 0140110, MF).

The HN inoculation group HNck-HNin (**Figure 4D**), a total of 104 GO term were significant enriched, compromising 58 BP, 32 CC, and 14 MF. The DEGs were significantly enriched in thylakoid (GO: 0009579, CC), thylakoid part (GO: 0044436, CC), photosynthetic membrane (GO: 0034357, CC), chloroplast thylakoid (GO: 0009534, CC), plastid thylakoid (GO: 0031976, CC), thylakoid membrane (GO: 0042651, CC), photosynthesis (GO: 0015979, BP), plastid part (GO: 0044435, CC), chloroplast thylakoid membrane (GO: 0009535, CC), plastid thylakoid membrane (GO: 0055035, CC), chloroplast part (GO: 0044434, CC), chloroplast (GO: 0009507, CC), photosynthesis, light harvesting (GO: 0009765, BP), photosynthesis, light reaction (GO: 0019684, BP), plastid (GO: 0009536, CC), photosystem (GO: 0009521, CC), chlorophyll binding (GO: 0016168, MF), pigment binding (GO: 0031409, MF), photosynthesis, light harvesting in photosystem I (GO: 0009768, BP), photosystem I (GO: 0009522, CC).

Among these enriched GO terms, LN inoculation group (LNck-LNin) and HN inoculation group (HNck-HNin) shared 50 significantly enriched GO terms, including 24 BP, 16 CC and 10 MF. he identified BP terms were primarily associated with photosynthesis, amino acid transport metabolism, and secondary metabolism. Examples of these BP terms include photosynthesis (GO: 0015979), amino acid transport (GO: 0006865), and secondary metabolic process (GO: 0019748). The CC terms mainly represented various components involved in photosynthesis such as chloroplasts, plastids, thylakoids, and cell membranes. Examples of these CC terms include chloroplast thylakoid (GO: 0009534), membrane part (GO: 0044425), and photosystem (GO: 0009521). Furthermore, the MF terms demonstrated a strong association with photosynthesis as well, encompassing chlorophyll binding (GO: 0016168), tetrapyrrole binding (GO: 0046906) and iron ion binding (GO: 0005506). Additionally, there was an enrichment in GO terms related to plant stress response such as oxidoreductase activity (GO: 0016491), cofactor binding (GO: 0048037) and monooxygenase activity (GO: 0004497).

Furthermore, both the LN inoculation group (LNck-LNin) and HN inoculation group (HNck-HNin) exhibited significant enrichment of specific Gene Ontology (GO) terms within their respective groups. In the low nitrate group, the most significantly enriched GO terms were associated with cellular membrane and its components, such as intrinsic component of membrane (GO: 0031224) and integral component of membrane (GO: 0016021), as well as defense response (GO: 0006952). Conversely, in the LNin-HNin inoculated group, there was a distinct enrichment of GO term related to generation of precursor metabolites and energy (GO: 0006091) (**Supplementary Table S6**). Overall, for each comparison group, the majority of enriched GO terms were linked to photosynthesis. We hypothesize that when supplied as a nitrogen source, varying concentrations of nitrate may impact rice photosynthesis and potentially play a crucial role in regulating resistance mechanisms in rice.

### KEGG enrichment

**Figure 5:**
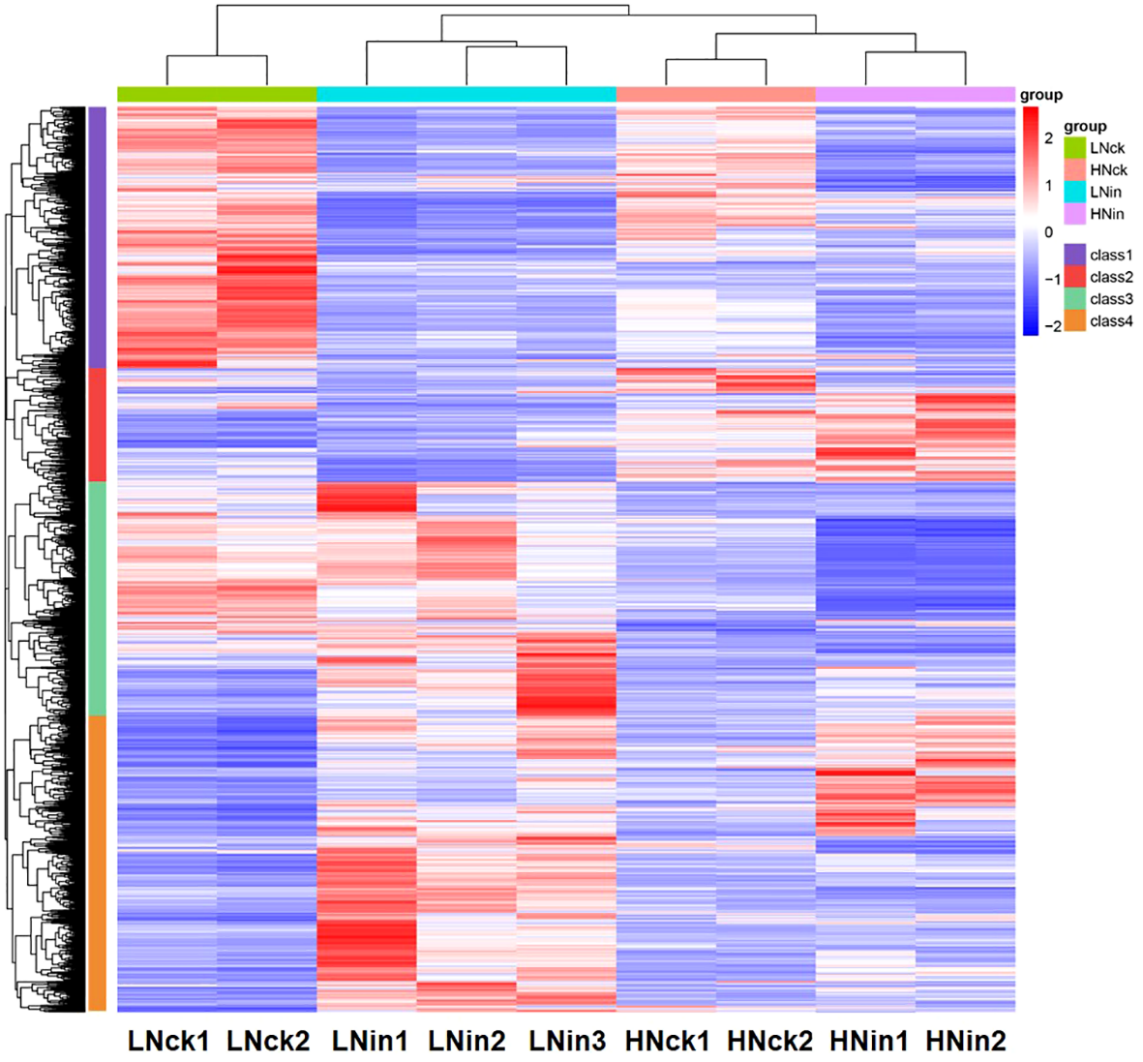
KEGG enrichment analysis of the DEGs.

Differential gene expression analysis was conducted for each comparison group, and the DEGs were subjected to KEGG pathway enrichment analysis. A significance threshold of *P*-value < 0.05 was applied to identify significantly enriched pathways. Overall, a total of 120 pathways exhibited enrichment across all groups, with 433 up-regulated DEGs and 653 down-regulated DEGs annotated to these pathways (**Supplementary Table S7**). Notably, there were 40 distinct pathways that demonstrated significant enrichment, encompassing annotations for 243 up-regulated DEGs and 341 down-regulated DEGs (**Figure 5**).

Plant-pathogen interactions (osa04626), known to be closely associated with rice’s resistance mechanism, exhibited significant enrichment in the inoculated groups (LNck-LNin, LNin-HNin). In the LNck-LNin cluster, 32 genes were up-regulated while 12 genes were down-regulated. Within the LNin-HNin cluster, there was an up-regulation of 10 genes and a down-regulation of 20 genes. Further analysis revealed that the up-regulated genes within the LNck-LNin cluster predominantly belonged to PTI.

In each group, we observed that the Photosynthesis - antenna proteins pathway (osa00196) was significantly enriched, which held true for LNck-HNck, HNck-HNin, and LNck-LNin. Notably, within both the LN inoculation and HN inoculation groups, the DEGs enriched in this pathway exhibited down-regulation. Antenna proteins are essential components involved in capturing light energy during photosynthesis. The light-harvesting complex (LHC), comprising Lhca and Lhcb subclasses responsible for light collection in PSI and PSII respectively, showed enrichment of down-regulated genes across both subclasses in both the LN and HN groups (**Figure S3**). These findings highlight the significant role of photosynthesis in elucidating the molecular mechanism underlying rice resistance to bacterial blight disease under nitrate nutrition.

### Identification of NPF family members involved in defense

**Figure 6:**
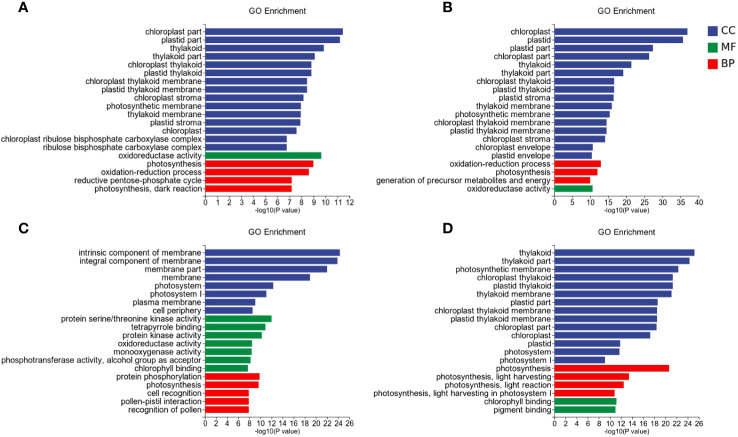
Heat maps depicting gene expression of resistance genes and NPF family members. **(A)** LNck-HNck. **(B)** LNin-Hnin. **(C)** HNck-Hnin. **(D)** HNck-Hnin.

In the previous phenotypic results, we found that increasing concentration of nitrate supply enhances rice resistance. In rice, the absorption and transport of nitrate nitrogen are mainly regulated by members of the NRT1/PTR (nitrate transporter 1/peptide transporter family, NPF) family (Leran et al., 2014). Therefore, in this study, we identified the NPF family members of DEGs in each group. We identified 1 upregulated member and 5 downregulated members in LNck-HNck, 7 upregulated members and 14 downregulated members in LNck-LNin, 3 upregulated members and 8 downregulated members in HNck-HNin, and 3 upregulated members and 5 downregulated members in LNin-HNin group (**Figure 6**). These family members exhibited different expression patterns with some showing differential expression across different comparison groups. For example, *OsNPF3.1* was upregulated in both LNck-HNck and LNin-HNin groups; *OsNPF4.1* is downregulated in both LNck-HNck and LNck-LNin groups; *OsNPF4.5*, *OsNPF5.13*, *OsNPF5.14*, *OsNPF5.24*, *OsNPF8.4*, *OsNPF8.13* and *OsNPF8.14* were down-regulated in both HNck-HNin and LNin-HNin groups; OsNPF6.5 and *OsNPF7.4* were downregulated in both LNck-LNin and LNin-HNin groups (**Figure 6**). Furthermore, there were cases where the differential expression pattern is opposite between different comparison groups such as *OsNPF7.1* which was up-regulated in LNck- HNck group but down-regulated in LNin- HNin group while the expression pattern of *OsNPF7.1* was opposite, it’s upregulated in LNin -HNin group and down regulated in LNck-LNin group (**Figure 6**). These results suggest that there may be certain NPF family members involved in response to pathogen invasion which require further discussion and validation.

### Verification with qRT-PCR

**Figure 7:**
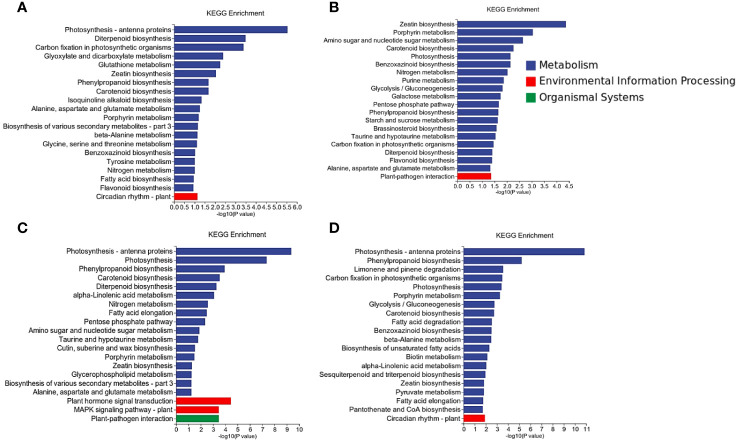
Quantitative RT-PCR validation of randomly selected genes. **(A)** LNck-HNck. **(B)** LNin-Hnin. **(C)** LNck-LNin. **(D)** HNck-HNin. Values are mean ± SD (n=3).

To validate the reliability of RNA-seq results, 14 different genes were randomly selected for qRT-PCR validation. *OsERF104*, *OsACS6*, *OsbHLH6*, *OsPsbR1*, *OsHSP71.1*, *OsBBTI1*, *OsTPS28*, *OsNRT2.4*, *OsNPF3.1*, *OsNPF7.4*, *OsNPF.7.11*, *OsNPF5.5*, *OsRING30*, and *OsMST2* were chosen to validate the RNA-seq results with qRT-PCR analysis using gene-specific primers. The qRT-PCR showed that these genes were consistent with the RNA-seq results (**Figure 7**).

## Discussion

### Differentially expressed genes in the comparison of inoculation under different nitrate concentrations

During the preliminary phenotypic investigation, we observed a significant influence of varying concentrations of nitrate supply on rice resistance to bacterial leaf blight. Notably, disease lesion length was significantly reduced when rice was exposed to the pathogen under high nitrogen (HN) supply, compared to low nitrogen (LN) supply. This finding suggests that HN nutrition can effectively enhance rice resistance against bacterial leaf blight. Specifically focusing on DEGs induced by inoculation under LN and HN supplies (LNck-LNin and HNck-HNin), we identified 279 DEGs commonly upregulated in both LN and HN groups, as along with 453 DEGs commonly downregulated in both groups. Additionally, apart from these shared regulations, certain genes exhibited opposite expression patterns between the two groups. Notably, 12 DEGs were upregulated in the HN group but downregulated in the LN group, while 27 DEGs showed contrasting regulation with upregulation in LN and downregulation in HN. Amongst these commonly upregulated genes, our attention was drawn towards *OsWRKY46* (*LOC_Os11g02480*) and *OsWRKY42* (*LOC_Os02g26430*), which have previously been reported to display heightened expression levels of a xyloglucan endotransglucosylase/hydrolase inhibitor factor (RIXI), resulting in enhanced resistance against blast fungus when overexpressed. The induction of RIXI is accompanied by PR gene activation along with increased hydrogen peroxide content and augmented antioxidant enzyme activity. Therefore, it is postulated that *OsWRKY46* may bolster rice resistance against pathogens through its role in promoting RIXI expression (Gao et al., 2020). In previous studies, *OsWRKY42* has been identified as a crucial regulatory factor that plays an important role in the resistance mechanism of rice against bacterial leaf blight disease. As a negative regulator in JA-mediated responses, *OsWRKY42* serves as a component of the *Xa21*-mediated pathway. Its expression is induced by both biotic and abiotic stresses such as pathogen invasion and salt stress. Knocking down *OsWRKY42* through RNAi reduces rice resistance to bacterial leaf blight (Ullah et al., 2023). Based on these findings, it is speculated that the WRKY transcription factor family, one of the most important transcription factor families in higher plants, plays a significant role in conferring resistance against bacterial leaf blight.

### NPF family members in differentially expressed genes

The NPF family, known as a reported transporter protein family involved in nitrate uptake and transport, has received considerable attention. In comparison to other transporter protein families, the NPF family exhibits a substantial number of members. *Arabidopsis thaliana* contains 53 NPF genes, while *Oryza Sativa* possesses 93 NPF genes (Leran et al., 2014; Wang et al., 2020; Li et al., 2021). A considerable portion of these family members has been analyzed and their biological functions elucidated. Notably, NPF family members play pivotal roles in diverse biological processes within rice plants. In this study, we identified differentially expressed genes belonging to the NPF family. *OsNPF3.1* exhibited upregulation under both low nitrogen conditions without any discernible difference between LN or HN groups alone (**Figure 6**). This observation suggests that *OsNPF3.1* specifically responds to alterations in nitrogen concentration or even nitrate concentration levels. Previous reports have indicated that two natural variation sites within *OsNPF3.1* are associated with nitrogen use efficiency (NUE), thereby influencing rice’s overall NUE and biomass production capacity (Yang et al., 2023). Combining our findings with previous analyses, it is plausible to propose that *OsNPF3.1* serves as a crucial gene regulating NUE independently of inoculation effects. Studies have demonstrated that overexpression of *OsNPF7.1* promotes nitrate absorption, while increasing the concentration of nitrate supply enhances plant biomass and yield. Conversely, knockdown of this gene through RNAi leads to opposite phenotypes (Huang et al., 2019). Based on these findings, *OsNPF7.1* is downregulated under LNck-LNin conditions, shows no significant difference in expression under HNck-HNin conditions, and is upregulated under LNin-HNin conditions. It can be speculated that inoculation may result in the downregulation of this gene, thereby impacting rice plant growth and reducing yield. However, when subjected to the same inoculated conditions with increased nitrate concentration supply, gene expression is upregulated, partially mitigating the negative effects caused by inoculation and resulting in improved resistance to high nitrogen compared to low nitrogen at the phenotype level. Interestingly, *OsNPF4.5*, *OsNPF5.13*, *OsNPF5.14*, *OsNPF5.24*, *OsNPF8.13*, *OsNPF8.14*, and *OsNPF8.4* exhibited downregulation in both the low nitrogen (LN) and high nitrogen (HN) inoculated groups; however, no differential expression was observed in the remaining two groups (**Figure 6**). This suggests that these genes are induced by inoculation but have not been cloned yet, requiring further comprehensive investigation. In addition to these NPF family members identified with a twofold difference in expression levels, there are also some stably highly expressed members with approximately 1.5-fold differences worth further attention; for instance, under HN nutrition conditions, consistent upregulation of expression upon inoculation is observed for *OsNPF5.5* (**Figure 6**).

### Relevance of Photosynthesis in Enhancing Plant Disease Resistance

Photosynthesis, a fundamental physiological process in plants, plays a crucial role throughout their lifecycle and is considered a key component of crop productivity for economic crops like rice. Previous studies have demonstrated that pathogen infection can lead to decreased photosynthetic activity due to active regulation during the plant’s immune response. The plant upregulates its immune reactions through the MAPK cascade pathway while simultaneously inhibiting photosynthesis to accumulate large amounts of reactive oxygen species (ROS) within chloroplasts to enhance its defense response. Therefore, photosynthesis not only regulates plant growth and development but also plays a significant role in plant defense responses (Kamoun et al., 2018). In our current study, we observed similar phenomena in rice after pathogen inoculation, with substantial gene responses related to photosynthesis mainly enriched for downregulation through KEGG and GO analyses. Specifically focusing on the down-regulated DEGs in HNck-HNin group and conducting GO enrichment analysis separately showed significant enrichment of these DEGs in KEGG pathways associated with photosynthesis-related processes (**Figures 4**, **5**). Similarly, when performing GO enrichment analysis on DEGs from LNin-HNin group, we also found significant enrichment primarily within components related to photosynthesis. It is apparent that pathogen inoculation under different nitrate concentrations has a considerable impact on rice’s photosynthetic activity and resistance mechanisms against bacterial blight.

### Differentially expressed genes involved in Plant Disease Resistance

Previous studies have identified a group of susceptible and resistant genes that play crucial roles in the disease resistance mechanism of rice, shedding light on the resistance mechanisms against diseases. Our phenotypic results demonstrate a significant decrease in lesion length after inoculation as nitrate supply concentration increases, indicating an enhancement in rice’s disease resistance. Differential expression analysis of resistant genes reveals specific responses under relatively low or high nitrate supply conditions, contributing to the enhanced disease resistance in rice. For instance, *OsRLR1* exhibits no differential expression under low nitrogen (LN) supply but is upregulated upon inoculation under high nitrogen (HN) conditions. Previous research has shown that this gene confers broad-spectrum resistance to bacterial leaf blight and blast diseases in rice; its overexpression enhances rice’s resistance to these pathogens, suggesting its positive regulatory role in defense response (Du et al., 2021). Conversely, *OsbHLH6* has been previously identified as a negative regulator of rice’s disease resistance; its overexpression leads to increased expression of JA-responsive genes and susceptibility to pathogenic bacteria. Interestingly, our current findings reveal that *OsbHLH6* is upregulated upon inoculation under HN conditions but downregulated under LN conditions (Meng et al., 2020). Regarding *OsSWEET14* which acts as a susceptible gene in white leaf blight disease in rice, CRISPR/Cas9-mediated knockout experiments have demonstrated enhanced resistance against this pathogen when *OsSWEET14* is knocked out (Hutin et al., 2015). We also observed an upregulation of its expression upon inoculation at both nitrate concentrations; however, the magnitude of upregulation was significantly higher under LN than HN conditions. These examples may demonstrate that differential expression of these key disease resistance genes in rice against bacterial leaf blight is attributed to variations in nitrate concentration supply, leading to divergent resistance outcomes between the two groups.

In this study, we observed a significantly higher number of DEGs induced by LNck-LNin compared to HNck-HNin. Furthermore, the upregulated DEGs in LNck-LNin were approximately 1.5 times more abundant than the downregulated ones, while the downregulated DEGs in HNck-HNin were approximately 1.5 times more prevalent than the upregulated ones. Additionally, there was also an increased presence of downregulated DEGs in LNin-HNin. This phenomenon may be attributed to distinct response mechanisms of rice to bacterial leaf blight under varying nitrate nutrition conditions. Under low nitrate nutrition conditions, rice might enhance its resistance to bacterial leaf blight by upregulating disease-resistant genes; however, under high nitrate nutrition conditions where rice already possesses strong resistance capabilities, it may not require further upregulation of these genes and instead even downregulate them as a preventive measure against overreaction. This parallels our immune system’s functioning - maintaining a balanced state when our body is healthy but becoming activated and producing stronger reactions when attacked by viruses or bacteria. However, prolonged hyperactivity of our immune system can lead to adverse reactions such as autoimmune diseases; hence achieving a balance between activation and inhibition is crucial for both humans and rice plants through gene expression regulation. This explanation represents one possible hypothesis requiring further research for confirmation.

## Data availability statement

The original contributions presented in the study are publicly available. This data can be found here: NCBI, PRJNA112983.

## Author contributions

XL: Data curation, Investigation, Methodology, Writing – original draft. SC: Data curation, Formal analysis, Investigation, Methodology, Validation, Writing – review & editing. CM: Data curation, Investigation, Validation, Writing – review & editing. HY: Formal analysis, Investigation, Writing – review & editing. QL: Investigation, Writing – review & editing. HJ: Data curation, Formal analysis, Writing – original draft. JC: Conceptualization, Data curation, Project administration, Supervision, Visualization, Writing – review & editing.

## Supplementary material

Supplementary Table 6 GO enrichment results.

Supplementary Table 7 KEGG enrichment results.
