# Supplementary material for: Transcriptome analysis of differentially expressed genes in rice seedling leaves under different nitrate treatments on resistance to bacterial leaf blight
Source: Front Plant Sci. 2024 Jul 4;15:1436912. doi: 10.3389/fpls.2024.1436912 (PMC11254694; doi:10.3389/fpls.2024.1436912)
Supplement: Supplementary file 8 [file Presentation_1.pptx]

## Slide 1
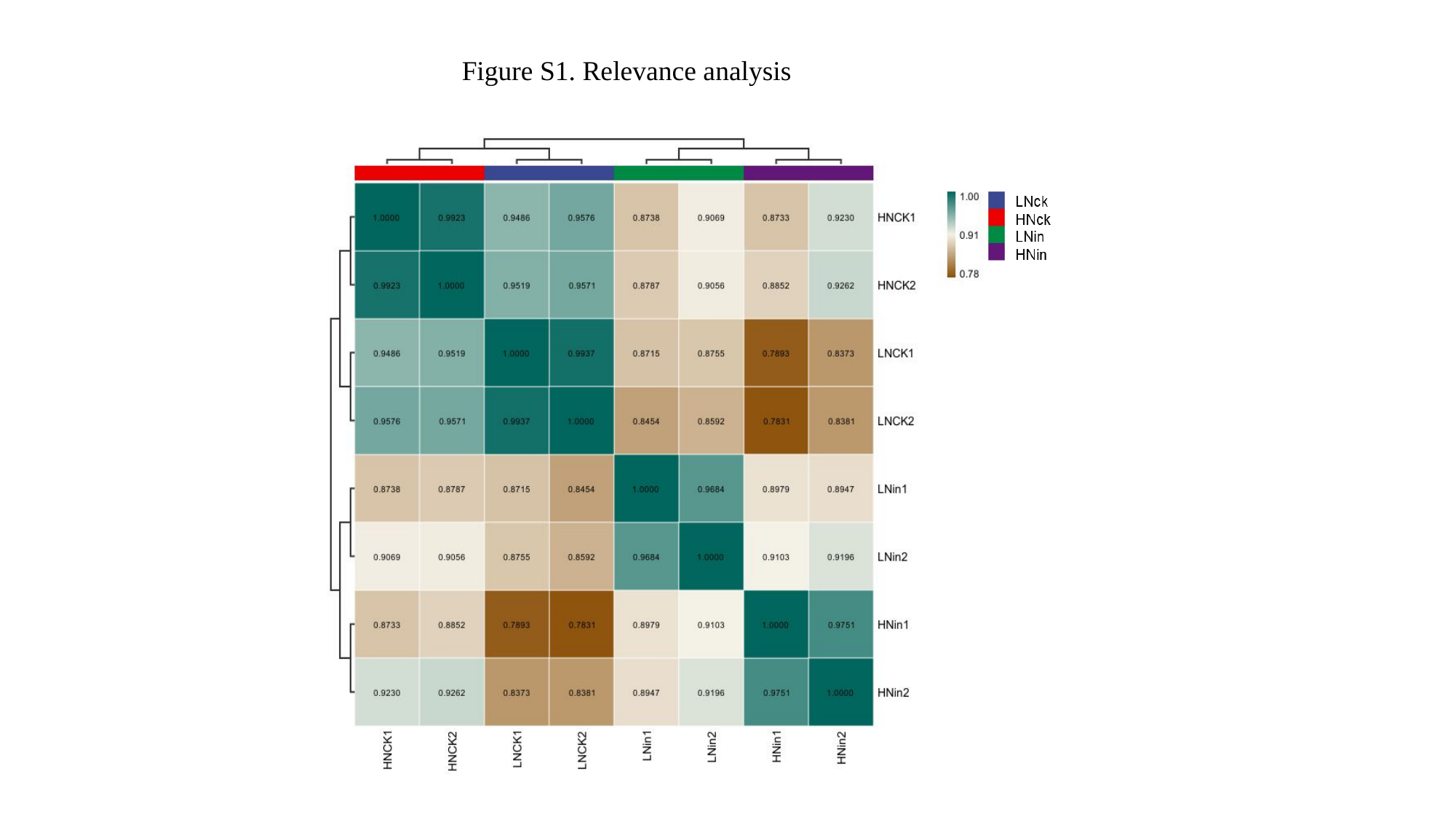

Figure S1. Relevance analysis

## Slide 2
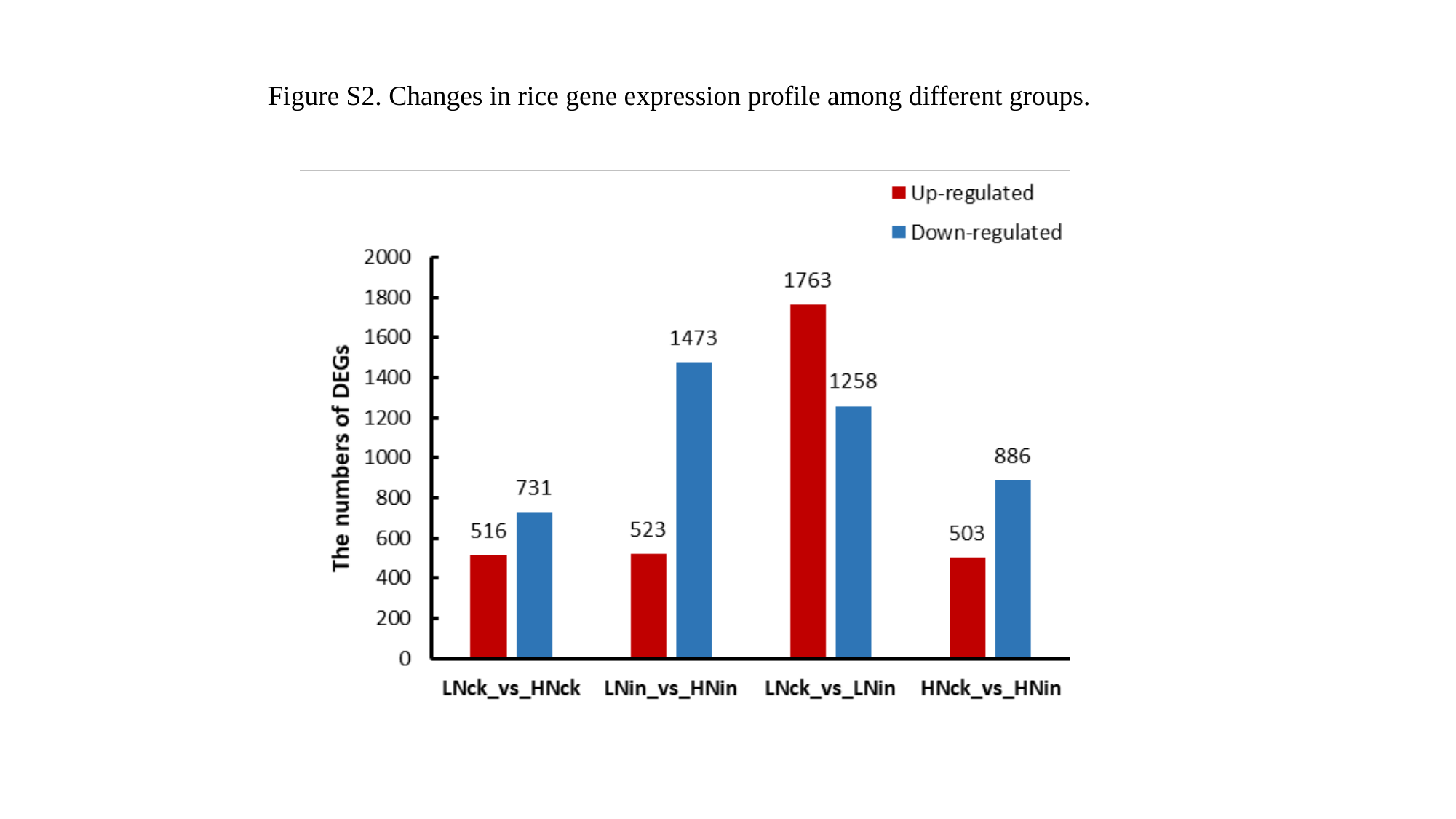

Figure S2. Changes in rice gene expression profile among different groups.

## Slide 3
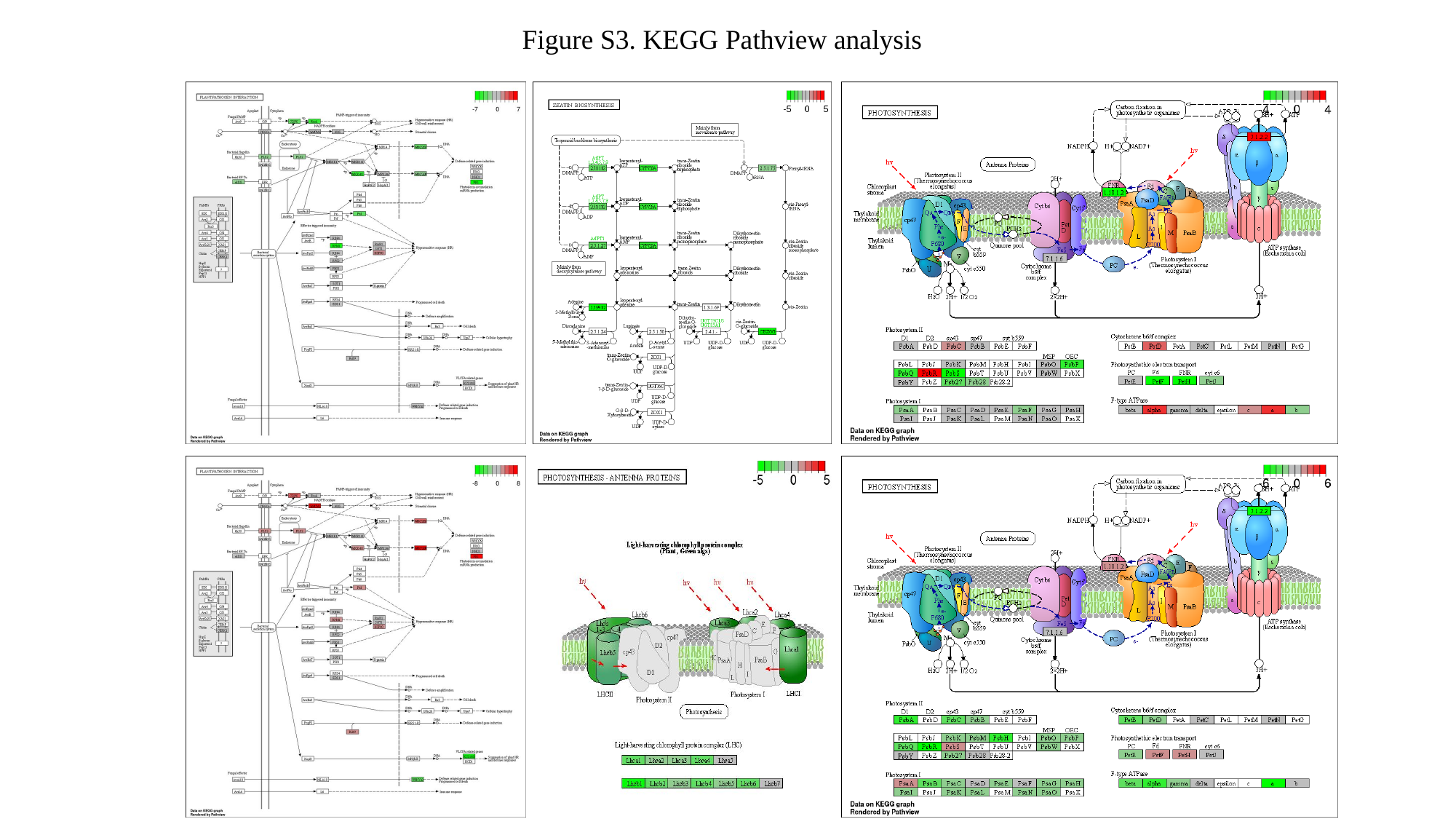

Figure S3. KEGG Pathview analysis
